# Leiomyosarcoma of the inferior vena cava as etiology of chronic abdominal pains – a case report

**DOI:** 10.1590/1677-5449.202101291

**Published:** 2022-09-19

**Authors:** Raíssa Campos D’Amico, Tamara Marques Ziliotto, Rayssa Marquesa Ávila, Stela Kremmer Bezerra Paes, José Sampaio, Giovanna Golin Guarinello, Jéssica Prado da Silva

**Affiliations:** 1 Santa Casa de Curitiba, Departamento de Cirurgia Vascular, Curitiba, PR, Brasil.

**Keywords:** leiomyosarcoma, vena cava, inferior, retroperitoneal neoplasms

## Abstract

Inferior vena cava leiomyosarcomas are rare tumors that account for less than 0.7% of all retroperitoneal leiomyosarcomas. They are more common in women and cause nonspecific chronic abdominal pain. In this report, we present the case of a 53-year-old female patient complaining of chronic nonspecific periumbilical abdominal pain with initial onset 8 months previously who was diagnosed with inferior vena cava leiomyosarcoma by computed tomography angiography. The patient was treated with complete resection of the tumor and reconstruction of the inferior vena cava with interposition of a Dacron prosthetic graft. The treatment considered the gold standard consists of complete surgical excision, because these tumors are resistant to chemotherapy and radiotherapy. The prognosis of these patients is closely related to early diagnosis. Therefore, it is very important that vascular and general surgeons know that this disease is a possible differential diagnosis of chronic abdominal pains.

## INTRODUCTION

Leiomyosarcoma of the inferior vena cava (IVC) is a retroperitoneal tumor that originates in the smooth muscle cells of tunica media of the vena cava wall and can grow intraluminally, extraluminally, or both.^1^^,^^2^ It is a very rare medical condition, with around 350 cases reported in the literature.^2^^,^^3^ Nonspecific abdominal pains are its most common clinical manifestation, because of the retroperitoneal location, and since it can mimic a series of abdominal diseases, it is rarely considered as initial differential diagnosis.^1^ The objective of this case report is to present leiomyosarcoma of the IVC as an etiology of chronic abdominal pains, shedding light on this disease as a differential diagnosis possibility in patients with nonspecific, long duration, abdominal pains. Knowledge of the disease is very important because early diagnosis is associated with lower risk of metastases, higher survival rates, and an increased possibility of cure.

This article was approved by the Research Ethics Committee, consolidated opinion no. 4.742.968 (CAAE 45143321.5.0000.0099).

## CASE REPORT

The patient was a 53-year-old female with systemic arterial hypertension, was taking 10 mg/day of enalapril, and had a 40 pack-years smoking habit. She had made multiple visits to an Urgent Care Center because of continuous abdominal pains in the periumbilical area, irradiating to the dorsal aspect, with onset 8 months previously, and associated with abdominal distension and weight loss of 3 kg over the period, but without nausea or vomiting. She reported being seen at the general surgery clinic and having laboratory tests and imaging exams for diagnostic investigation, but at presentation had not been diagnosed definitively. She sought care at the Urgent Care Center because of the extreme intensity of her abdominal pains, which were refractory to analgesics prescribed for home use. Physical examination found her abdomen rounded, slightly distended, tympanic to percussion, and painful on superficial and deep palpation in the periumbilical region and flanks, without visceromegaly or signs of peritoneal irritation. The remainder of the examination was unremarkable. The tests and examinations performed previously were as follows: ultrasound of the entire abdomen, performed 8 months previously, showing a nodule in the region between the aorta and cava measuring 40 x 24 mm, suggestive of lymphadenomegaly; computed tomography without contrast, performed 15 days earlier, showing calcifed granuloma in liver segment IV, with no other abnormalities; and colonoscopy and upper digestive endoscopy, with no findings that would explain the patient’s condition.

**Figure 1:**
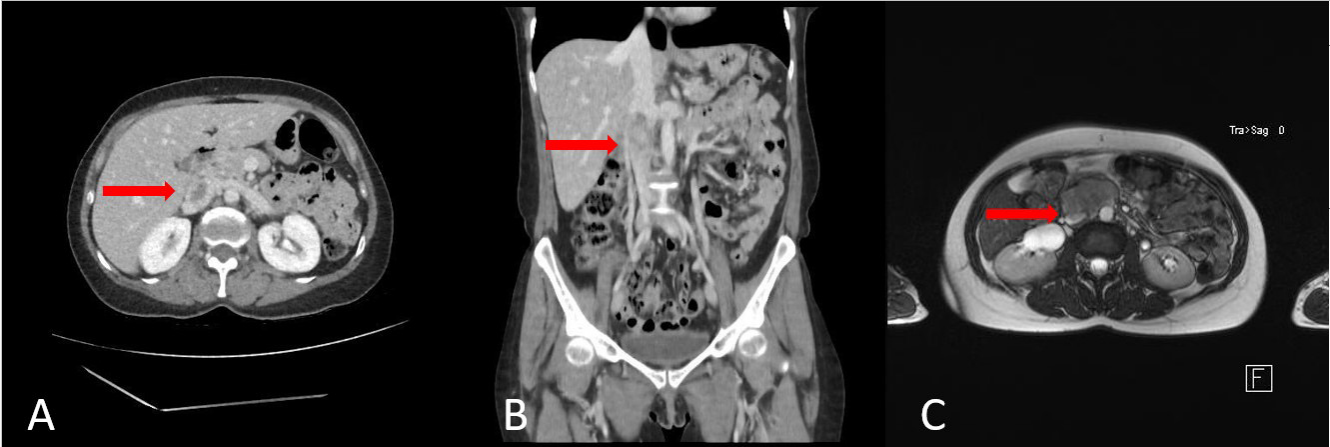
(A) and (B) Computed tomography angiography of the abdomen and pelvis in axial and coronal views, respectively; (C) Axial magnetic resonance in T2. Red arrows indicate the inferior vena cava leiomyosarcoma.

Computed tomography with contrast was ordered, showing a mass with lobulated outlines and heterogeneous density and intravenous contrast uptake in the retroperitoneal region between the aorta and cava. After admission, computed tomography angiography (CTA) and magnetic resonance of the abdomen and pelvis were performed, both showing an expansive lesion embedded in the IVC, with an anterior exophytic component, with focal cold areas suggestive of necrosis, with no contact with kidneys or adrenal glands, displacing the duodenum anteriorly, and measuring 93 x 54 x 36 mm (craniocaudal x transverse x anteroposterior). The cranial portion was completely intraluminal and at the level at which the renal veins drain to the IVC, while the caudal portion was extraluminal and extended to the level of the aortic bifurcation, which was suggestive of a leiomyosarcoma of the IVC (Figure 1).

**Figure 2:**
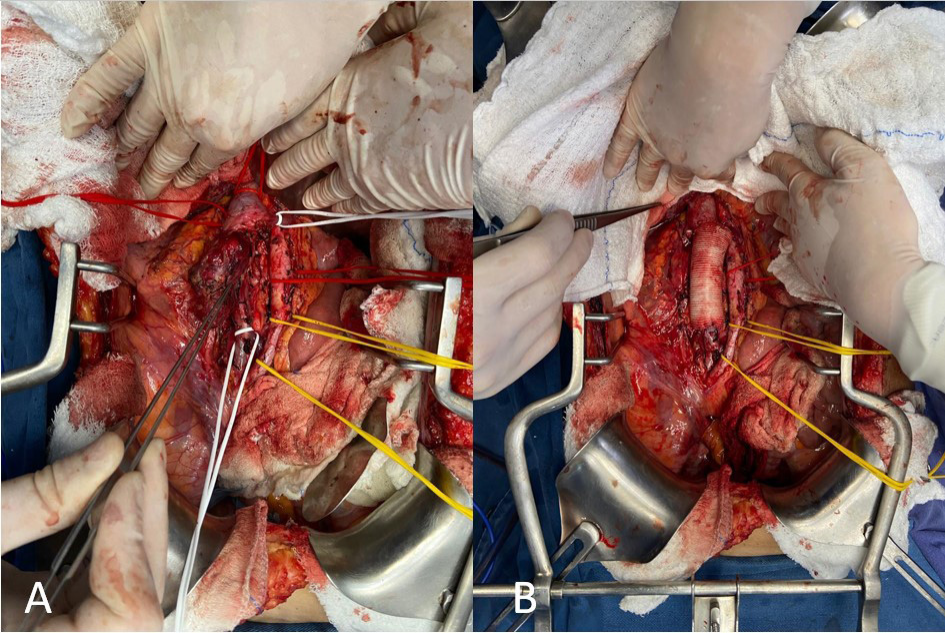
(A) Leiomyosarcoma of the inferior vena cava after dissection; (B) Reconstruction of the inferior vena cava with interposition of a Dacron graft; final result.

**Figure 3:**
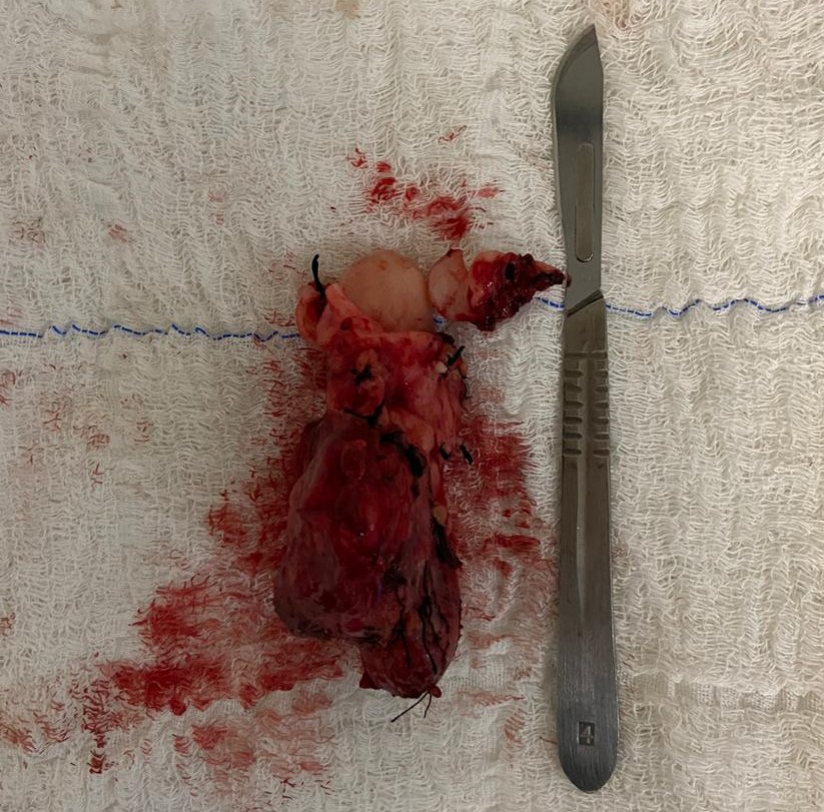
Surgical specimen after resection en bloc.

**Figure 4:**
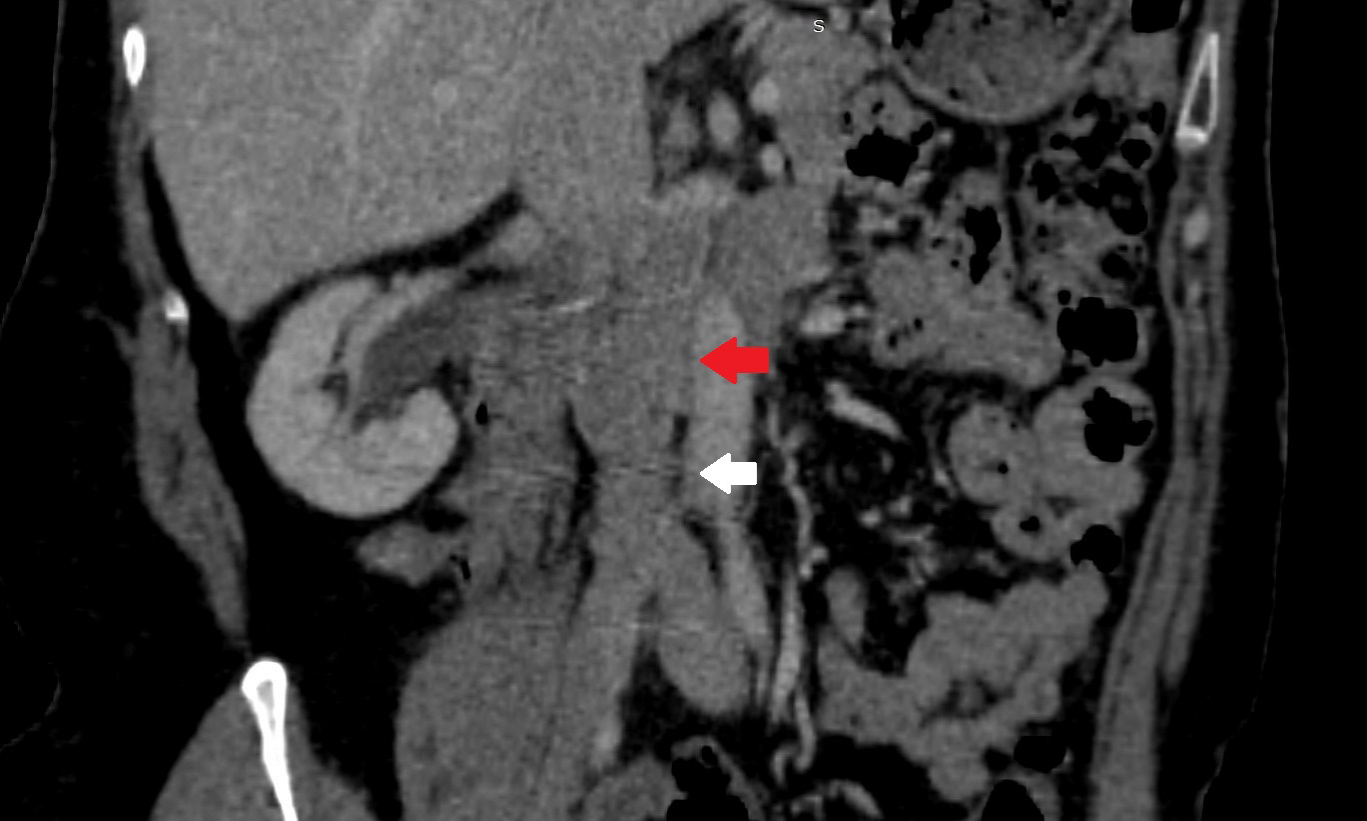
Coronal computed tomography angiography of the abdomen and pelvis in the fifth postoperative month. Red arrow indicates the Dacron graft; white arrow indicates the distal anastomosis with the inferior vena cava.

A joint vascular surgery and general surgery team performed a surgical procedure via transabdominal access. The IVC mass was identified, starting below the outflow of the left renal vein, with a length of approximately 8 cm, ending above the bifurcation of the common iliac veins, but without invading the infrarenal abdominal aorta. The team proceeded to dissection, removing the retroperitoneal tumor en bloc by complete circumferential resection of the IVC section containing it and then reconstructed the IVC by interposition of a 24 mm tubular prosthetic Dacron graft, with proximal anastomosis, including the renal vein and proximal IVC, and distal anastomosis, including the distal IVC (Figure 2). Postoperative recovery in the intensive care unit was uneventful. Anatomopathological examination of the specimen (Figure 3) revealed a mesenchymal spindle cell tumor with free margins, while immunohistochemistry confirmed a diagnosis of high grade leiomyosarcoma of the IVC. After confirmation of the diagnosis, the patient was referred for postoperative follow-up to the clinical oncology team, which detected pulmonary metastases and started palliative chemotherapy, with docetaxel and gemcitabine. The patient was discharged from the vascular surgery outpatients clinic after 5 months of follow-up, asymptomatic and with imaging exam within normal limits (Figure 4).

## DISCUSSION

Retroperitoneal leiomyosarcomas are rare tumors that account for less than 5% of all connective tissue tumors. Leiomyosarcomas originating in vascular tissue are even rarer, accounting for around 0.7% of cases. They originate in the smooth muscle cells of the tunica media of the vessel wall, the vascular structure they most often involve is the IVC, and they are more common among women over the age of 50.^3^^-^5

Classically, as described in our case report, these tumors are diagnosed late, because they occupy the retroperitoneal space and thus take a long time to provoke symptoms. When symptoms do emerge, they generally manifest through nonspecific conditions provoked by compression of adjacent structures, which can cause pain with uncertain location, making diagnosis more difficult.^3^^,^^6^ This is why they tend to be diagnosed at advanced stages and are therefore associated with poor prognosis.^3^^,^^4^^,^^6^

Symptoms vary depending on the anatomic region involved, so these leiomyosarcomas can be classified into three types. The first is when the infrarenal portion of the IVC is involved (type I), as in the case reported here, and accounts for 36% of cases. In these cases, clinical presentation is through edema of the lower limbs, deep venous thrombosis, and abdominal pain and distension. The second type, accounting for approximately 44% of cases, encompasses cases in which the tumor develops between the outflow of the renal vein and the hepatic vein (type II) and tends to involve impairment of renal function with nephrotic syndrome and abdominal pains. Finally, tumors that develop above the emergence of the hepatic vein (type III) are the rarest, accounting for only 20% of cases, and clinical presentation may include pulmonary thromboembolism (PTE) and Budd-Chiari syndrome, with hepatomegaly, ascites, and/or jaundice.^3^^,^^5^^,^^7^

Nowadays, as imaging exam technology has improved, these tumors are diagnosed earlier and, as a consequence, may be treated with the prospect of achieving a cure. Nevertheless, metastases can still occur, with predominantly hematogenic dissemination, primarily involving the lungs, liver, and bones.^3^^,^^5^

The examinations used for diagnosis are CTA, magnetic resonance angiography, and venography of the cava. In addition to diagnosis, the first two examinations also reveal invasion of adjacent structures by the tumor and its extension and location, enabling preoperative planning.^5^^,^^8^

The gold standard treatment is complete surgical excision, since these tumors are resistant to both chemotherapy and radiotherapy. Prognosis is directly related to sufficient resection with disease-free margins, both macroscopically and microscopically,^5^^,^^6^^,^^8^ and, in some cases, such as those in which there is involvement of the supra-hepatic segment, partial resection of the liver may also be needed.^8^

Countless different surgical strategies can be employed, depending on the location and size of the tumor. Total circumferential resection of the IVC should be preferred whenever possible, performing reconstruction with polytetrafluoroethylene (PTFE) or Dacron grafts – there is no consensus in the literature on the best type of synthetic graft material.^9^ Another option is venoplasty with primary IVC repair with bovine pericardium or prosthetic graft; but this technique has a reduced potential for achieving free margins. Primary repair without full circumferential resection should only be considered for small tumors.^5^^,^^7^

Ligature of the IVC without reconstruction can be considered in around 20% of cases of infrarenal leiomyosarcoma, when there is a rich network of collaterals to guarantee maintenance of venous return.^5^^,^^7^ Patients subjected to ligature without IVC reconstruction exhibit more severe edema during the postoperative period, but are at lower risk of developing PTE. In contrast, primary or prosthetic repair involves a low risk of developing postoperative edema, but is associated with a higher risk of PTE.^10^

Another situation that can present a surgical challenge is involvement of the right renal vein by the tumor. When it must be removed surgically and there is no possibility of reconstruction, a right nephrectomy should be considered. Simple ligature of the vein is not considered safe because of the associated risk of congestion and kidney failure. In general, the left renal vein is longer and has more collaterals to ensure venous return, and so ligature still permits preservation of the left kidney. However, whenever possible, reconstruction with a prosthetic IVC graft is recommended, with reimplantation of the renal vein by end-to-end anastomosis.^5^^,^^7^

Chemotherapy and radiotherapy are controversial for treatment of IVC leiomyosarcomas, because these tumors are usually resistant to both treatments, so they are generally only used in metastatic cases without indications for surgical treatment. However, few favorable results are observed, without impact on long term patient survival or reduction of local recurrence.^7^^,^^8^^,^^10^

The prognosis of patients with IVC leiomyosarcoma is completely dependent on the size of the tumor at diagnosis. Obviously, factors such as metastases worsen prognosis, as do compromised margins after surgical resection.^5^ It is known that the factor with greatest influence on patient survival is R0 surgical resection.^8^ Overall survival of patients with completely resected leiomyosarcomas with ample margins was 56% at 5 years and 47% at 10 years.^10^

Since the prognosis of these patients is intimately related with early diagnosis, it is extremely important that vascular surgeons and general surgeons know about this disease when conducting differential diagnosis of chronic and nonspecific abdominal pains.
