# Supplementary material for: Leiomyosarcoma of the inferior vena cava as etiology of chronic abdominal pains – a case report
Source: J Vasc Bras. 2022 Sep 19;21:e20210129. doi: 10.1590/1677-5449.202101291 (PMC9499726; doi:10.1590/1677-5449.202101291)
Supplement: Translation

# Translation: Leiomiossarcoma de veia cava inferior como etiologia para dor abdominal crônica – um relato de caso

**DOI:** 10.1590/1677-5449.202101292

**Authors:** Raíssa Campos D’Amico, Tamara Marques Ziliotto, Rayssa Marquesa Ávila, Stela Kremmer Bezerra Paes, José Sampaio, Giovanna Golin Guarinello, Jéssica Prado da Silva

**Affiliations:** 1 Santa Casa de Curitiba, Departamento de Cirurgia Vascular, Curitiba, PR, Brasil.

**Keywords:** leiomiossarcoma, veia cava inferior, neoplasias retroperitoneais

## Abstract

Os leiomiossarcomas de veia cava inferior são tumores raros, que representam menos de 0,7% de todos os leiomiossarcomas retroperitoneais. Eles são mais comuns em mulheres e causam quadros inespecíficos de dor abdominal crônica. Neste relato, apresentamos um caso de paciente do sexo feminino, de 53 anos de idade, com queixa de dor abdominal crônica periumbilical inespecífica com evolução há 8 meses, diagnosticada com leiomiossarcoma de veia cava inferior por angiotomografia computadorizada. A paciente foi tratada com ressecção completa do tumor e reconstrução da veia cava inferior, com interposição de prótese de dácron. O tratamento considerado padrão-ouro consiste na excisão cirúrgica completa, visto que esses tumores são resistentes a quimioterapia e radioterapia. O prognóstico desses pacientes está intimamente relacionado com a precocidade do diagnóstico, e, por isso, é de grande relevância o conhecimento dessa doença como diagnóstico diferencial de dor abdominal crônica e inespecífica por cirurgiões vasculares e cirurgiões gerais.

## INTRODUÇÃO

O leiomiossarcoma de veia cava inferior (VCI) é um tumor retroperitoneal com origem nas células musculares lisas da camada média da parede da veia cava, podendo ter crescimento intraluminal, extraluminal ou combinado^1^^,^^2^. Tal condição médica é bastante rara, com cerca de 350 casos reportados na literatura^2^^,^^3^. Devido à localização retroperitoneal, sua manifestação clínica mais frequente é a dor abdominal inespecífica, podendo mimetizar uma série de doenças abdominais e sendo raramente considerada como diagnóstico diferencial inicial^1^. O objetivo deste relato de caso é apresentar o leiomiossarcoma de VCI como etiologia de dor abdominal crônica, trazendo à tona essa doença como possibilidade de diagnóstico diferencial em pacientes com dores abdominais inespecíficas e de longa duração. O conhecimento dessa doença é de grande relevância, uma vez que, com o diagnóstico precoce, há menores riscos de metástases, maior taxa de sobrevida e maior possibilidade de cura.

O presente artigo foi aprovado pelo Comitê de Ética em Pesquisa, sob o parecer consubstanciado n.º 4.742.968 (CAAE 45143321.5.0000.0099).

## RELATO DE CASO

Tratava-se de uma paciente do sexo feminino, com 53 anos de idade, portadora de hipertensão arterial sistêmica, em uso de enalapril 10 mg/dia e tabagista (40 maços/ano). Ela apresentava história de múltiplas consultas em pronto atendimento devido à dor abdominal contínua, com início há 8 meses, de localização periumbilical, irradiada para dorso, associada a distensão abdominal e perda ponderal de 3 kg no período, sem náuseas ou vômitos. Relatava acompanhamento médico em ambulatório de cirurgia geral, com realização de exames complementares para elucidação diagnóstica, mas, até então, sem diagnóstico definido. A paciente procurou serviço de pronto atendimento devido à grande intensidade da dor abdominal, refratária a analgesia prescrita para uso domiciliar. No exame físico, evidenciou-se abdome globoso, pouco distendido e timpânico à percussão, doloroso à palpação superficial e profunda em região periumbilical e flancos, sem visceromegalias ou sinais de irritação peritoneal. O restante do exame não apresentou alterações dignas de nota. Os exames complementares prévios foram os seguintes: ultrassom de abdome total, realizado 8 meses antes, demonstrando presença de nódulo na região interaortocaval medindo 40 × 24 mm, sugestivo de linfonodomegalia; tomografia computadorizada sem contraste, realizada 15 dias antes, com presença de granuloma calcificado no segmento hepático IV, sem demais alterações; e colonoscopia e endoscopia digestiva alta, sem alterações que justificassem o quadro.

Foi solicitada tomografia computadorizada com contraste, que evidenciou massa de contornos lobulados com densidade e realce heterogêneo pelo meio de contraste endovenoso, localizado na região retroperitoneal interaortocaval. Após internamento, foi solicitada angiotomografia computadorizada (ATC) e ressonância magnética de abdome e pelve, ambas mostrando presença de lesão expansiva embutida na VCI, com componente exofítico anterior, com focos hipocaptantes sugestivos de necrose, sem contato com rins e adrenais, deslocando duodeno anteriormente, medindo 93 × 54 × 36 mm (craniocaudal × transverso × anteroposterior). A porção mais cranial era completamente intraluminal e ao nível da desembocadura das veias renais, e a porção mais caudal era extraluminal e se estendia até o nível da bifurcação aórtica, o que sugeria leiomiossarcoma de VCI (Figura 1).

**Figura 1:** (A) e (B) Angiotomografia computadorizada de abdome e pelve em corte axial e coronal, respectivamente; (C) Ressonância magnética em corte axial em T2. Seta vermelha demonstrando leiomiossarcoma de veia cava inferior.

Foi realizada abordagem cirúrgica conjunta da cirurgia vascular e cirurgia geral, através de acesso transabdominal. Identificou-se presença de massa em VCI, iniciando-se abaixo da desembocadura da veia renal esquerda, com extensão por aproximadamente 8 cm, terminando acima da bifurcação das veias ilíacas comuns, sem invasão da aorta abdominal infrarrenal. Foi realizada a dissecção, com retirada em bloco do tumor retroperitoneal através de ressecção circunferencial completa da VCI contendo a massa, e feita a reconstrução da VCI através da interposição de prótese de dácron tubular de 24 mm, com anastomose proximal, incluindo a veia renal e a VCI proximal, e anastomose distal, incluindo a VCI distal (Figura 2). O pós-operatório ocorreu sem intercorrências, em unidade de terapia intensiva. O exame anatomopatológico da peça (Figura 3) demonstrou neoplasia mesenquimal fusocelular, com margens livres; a imunohistoquímica confirmou diagnóstico de leiomiossarcoma de VCI de alto grau. Após diagnóstico confirmado, a paciente iniciou acompanhamento com a equipe da oncologia clínica; no seguimento pós-operatório, foram identificadas metástases pulmonares e foi iniciada quimioterapia de cunho paliativo, com docetaxel e gencitabina. A paciente recebeu alta ambulatorial da cirurgia vascular após 5 meses de acompanhamento, assintomática e com exame de imagem dentro da normalidade (Figura 4).

**Figura 2:** (A) Leiomiossarcoma de veia cava inferior após dissecção; (B) Reconstrução de veia cava inferior com interposição de prótese de dácron; resultado final.

**Figura 3:** Peça cirúrgica após ressecção em bloco.

**Figura 4:** Angiotomografia computadorizada de abdome e pelve em corte coronal no quinto mês pós-operatório. Seta vermelha demonstrando a prótese de dácron; seta branca indicando área de anastomose distal com a veia cava inferior.

## DISCUSSÃO

Os leiomiossarcomas retroperitoneais são tumores raros que correspondem a menos de 5% de todos os tumores de tecido conjuntivo. Os leiomiossarcomas com origem em tecido vascular são ainda mais raros, representando cerca de 0,7% dos casos. Eles têm origem nas células musculares lisas da camada média da parede do vaso, sendo a VCI a estrutura vascular mais acometida, e são mais frequentes em mulheres após os 50 anos^3^^-^5.

Classicamente, conforme apresentado em nosso relato de caso, são tumores de diagnóstico tardio justamente pelo fato de acometerem o retroperitônio, levando períodos prolongados de tempo para gerar sintomas. Quando se iniciam os sintomas, em geral, manifestam-se por meio de quadros inespecíficos pela compressão de estruturas adjacentes, o que pode causar dor de localização inespecífica e dificultar o diagnóstico^3^^,^^6^. Por tal motivo, costumam ser diagnosticados já em estágios avançados e, por isso, estão associados a prognóstico reservado^3^^,^^4^^,^^6^.

Os sintomas variam conforme a área anatômica acometida, o que permite a classificação do leiomiossarcoma em três tipos. O primeiro é quando há acometimento da porção infrarrenal da VCI (tipo I), como na situação relatada, que corresponde a 36% dos casos. Nesse caso, o quadro clínico manifesta-se através de edema de membros inferiores, trombose venosa profunda, dor e distensão abdominal. O segundo tipo ocorre quando o tumor se desenvolve entre a desembocadura da veia renal e veia hepática (tipo II), aproximadamente 44% dos casos, e tende a ocorrer comprometimento da função renal com ocorrência de síndrome nefrótica e também dor abdominal. Por fim, a lesão que cresce acima da emergência da veia hepática (tipo III), a mais rara, representa apenas 20% dos casos; nesse caso, a clínica pode ser de tromboembolismo pulmonar (TEP) e síndrome de Budd-Chiari, associando hepatomegalia, ascite e icterícia^3^^,^^5^^,^^7^.

Atualmente, com o avanço tecnológico dos métodos complementares de imagem, esses tumores têm sido mais diagnosticados e, consequentemente, tratados com intenção curativa. Ainda assim, metástases podem ocorrer, com disseminação prioritariamente hematogênica, acometendo principalmente pulmão, fígado e ossos^3^^,^^5^.

Os exames usados para diagnóstico consistem em ATC, angiorressonância magnética e venografia de cava. Além do diagnóstico, os dois primeiros exames permitem a determinação de invasão de estruturas adjacentes, extensão e localização do tumor, permitindo o planejamento pré-operatório^5^^,^^8^.

O tratamento considerado padrão-ouro consiste na excisão cirúrgica completa, dado que esses tumores são resistentes a quimioterapia e radioterapia. O prognóstico está diretamente relacionado com a ressecção ampla com bordas macroscópicas e microscópicas livres de doença^5^^,^^6^^,^^8^, e, em alguns casos, como no envolvimento do segmento supra-hepático, pode ser necessária ressecção hepática parcial associada^8^.

Inúmeras estratégias cirúrgicas podem ser empregadas, a depender da localização e extensão tumoral. Deve ser priorizada, sempre que possível, a ressecção circunferencial total da VCI com reconstrução com prótese de politetrafluoretileno (PTFE) ou dácron – não há consenso na literatura sobre o melhor tipo de enxerto sintético a ser usado^9^. Outra opção é a venoplastia com reparo primário da VCI, com pericárdio bovino ou prótese; essa técnica, porém, tem menor potencial de alcançar margens livres. O reparo primário sem ressecção circunferencial completa deve ser considerado apenas em casos de tumores pequenos^5^^,^^7^.

A ligadura sem reconstrução da VCI pode ser considerada em cerca de 20% dos casos de leiomiossarcoma infrarrenal, quando há presença de extensa rede de colaterais que garantem a manutenção do retorno venoso^5^^,^^7^. Os pacientes submetidos à ligadura sem reconstrução de VCI apresentaram edema mais grave no pós-operatório, no entanto, com menor risco de desenvolver TEP. Já o uso de reparo primário ou próteses tem baixo risco de desenvolver edema pós-operatório. No entanto, associa-se a um maior risco de TEP^10^.

Outra situação que pode representar um desafio cirúrgico consiste no envolvimento da veia renal direita pelo tumor. Quando ela precisa ser removida cirurgicamente e não há possibilidade de reconstrução, deve ser considerada a nefrectomia direita associada. Não é considerada segura a simples ligadura da veia, pelo risco de congestão e falência renal associada. Em geral, a veia renal esquerda é mais longa e tem mais colaterais que garantem o retorno venoso, e, por isso, sua ligadura permite a preservação do rim esquerdo. Entretanto, sempre que possível, recomenda-se a reconstrução com prótese da VCI, com reimplante da veia renal com anastomose terminoterminal^5^^,^^7^.

A quimioterapia e a radioterapia são temas controversos no tratamento dos leiomiossarcomas de VCI, porque esses tumores usualmente são resistentes a ambas terapias, sendo aplicadas apenas em casos metastáticos sem indicação de tratamento cirúrgico. Porém, poucos resultados favoráveis foram encontrados, sem impacto na sobrevida dos pacientes em longo prazo ou redução de recorrência local^7^^,^^8^^,^^10^.

O prognóstico de pacientes com leiomiossarcoma de VCI é completamente dependente da extensão do tumor no diagnóstico. Claramente, fatores como metástases comprometem o prognóstico, bem como margens comprometidas após ressecção cirúrgica^5^. Sabe-se que o maior influenciador na sobrevida do paciente é a ressecção cirúrgica R0^8^. A sobrevida geral de pacientes com leiomiossarcomas completamente ressecados com margem ampla foi de 56% em 5 anos e de 47% em 10 anos^10^.

Uma vez que o prognóstico desses pacientes está intimamente relacionado com a precocidade do diagnóstico, é de grande relevância o conhecimento dessa doença no diagnóstico diferencial de dor abdominal crônica e inespecífica por cirurgiões vasculares e cirurgiões gerais.
